# Modulation of T-Cell-Dependent Humoral Immune Response to Influenza Vaccine by Multiple Antioxidant/Immunomodulatory Micronutrient Supplementation

**DOI:** 10.3390/vaccines12070743

**Published:** 2024-07-04

**Authors:** Biljana Bufan, Nevena Arsenović-Ranin, Irena Živković, Ivana Ćuruvija, Veljko Blagojević, Luka Dragačević, Ana Kovačević, Jelena Kotur-Stevuljević, Gordana Leposavić

**Affiliations:** 1 Department of Microbiology and Immunology, Faculty of Pharmacy, University of Belgrade, 11221 Belgrade, Serbia; bbiljana@pharmacy.bg.ac.rs (B.B.); nevenaar@pharmacy.bg.ac.rs (N.A.-R.); 2 Department of Research and Development, Institute of Virology, Vaccines and Sera “Torlak”, 11221 Belgrade, Serbia; z92irena@gmail.com (I.Ž.); ivanajakovljev1@gmail.com (I.Ć.); veljko.blagojevic1988@gmail.com (V.B.); ldragacevic@torlak.rs (L.D.); 3 Department for Virology Control, Institute of Virology, Vaccines and Sera “Torlak”, 11221 Belgrade, Serbia; afilipovic@torlak.rs; 4 Department of Medical Biochemistry, Faculty of Pharmacy, University of Belgrade, 11221 Belgrade, Serbia; jelena.kotur@pharmacy.bg.ac.rs; 5 Department of Pathobiology, Faculty of Pharmacy, University of Belgrade, 11221 Belgrade, Serbia

**Keywords:** antioxidant supplementation, influenza vaccine, antibody response, germinal center reaction

## Abstract

Notwithstanding prevalence gaps in micronutrients supporting immune functions, the significance of their deficits/supplementation for the efficacy of vaccines is underinvestigated. Thus, the influence of supplementation combining vitamins C and D, zinc, selenium, manganese, and N-acetyl cysteine on immune correlates/surrogates of protection conferred by a quadrivalent influenza vaccine (QIV) in mice was investigated. The supplementation starting 5 days before the first of two QIV injections given 28 days apart increased the serum titres of total and neutralizing IgG against each of four influenza strains from QIV. Accordingly, the frequencies of germinal center B cells, follicular CD4+ T helper (Th) cells, and IL-21-producing Th cells increased in secondary lymphoid organs (SLOs). Additionally, the supplementation improved already increased IgG response to the second QIV injection by augmenting not only neutralizing antibody production, but also IgG2a response, which is important for virus clearance, through favoring Th1 differentiation as indicated by Th1 (IFN-γ)/Th2 (IL-4) signature cytokine level ratio upon QIV restimulation in SLO cell cultures. This most likely partly reflected antioxidant action of the supplement as indicated by splenic redox status analyses. Thus, the study provides a solid scientific background for further research aimed at repurposing the use of this safe and inexpensive micronutrient combination to improve response to the influenza vaccine.

## 1. Introduction

According to the World Health Organization (WHO), there are one billion cases of seasonal influenza worldwide each year, of which three–five million are severe cases leading to approximately 650,000 deaths per year [1]. The most effective way to prevent influenza infection is through vaccination [2]. The most used seasonal influenza vaccines are inactivated influenza virus vaccines administered through the intramuscular route [3]. Current seasonal influenza vaccines are prepared in line with the WHO recommendations and contain two influenza A viruses (H1N1 and H3N2) and one (in the trivalent influenza vaccine) or two [in quadrivalent influenza vaccine (QIV)] influenza B viruses from each lineage (Victoria and Yamagata) [4]. It is noteworthy that these vaccines have highly variable efficacy that ranges between 10% and 60% [5,6], being particularly low in certain vulnerable groups, such as the elderly, whose proportion of the general population is constantly increasing [7]. This is consistent with data indicating that the elderly have diminished antibody responses to vaccines, including the seasonal influenza vaccine [8]. Given that seasonal influenza vaccination campaigns are a major investment for countries and governments, it is clear that it is desirable to improve/maximize the effectiveness of the vaccine, not only from the global health point of view but also for economic reasons. In this context, it is important to identify correlates (immune response responsible for and interrelated with protection) and surrogates (immune response that substitute the true immunologic correlate of protection, which is unknown or not easily measurable) of protection against influenza [9]. Thus, it is noteworthy that they may be used instead of clinical outcomes, not only to evaluate the consistency of vaccine production but also to estimate the susceptibilities of individuals and populations after vaccination to the infection [10]. In adults, serum influenza-specific IgG antibody titre, particularly neutralizing IgG antibody titre, is commonly used as an indicator of efficacy of influenza vaccine, and a correlate of the protection against subsequent infections [9,10,11]. However, according to statistical analysis, CD4+ cell responses, crucial for Ig somatic hypermutation in B cells and generation of high-affinity, antibody-producing plasma cells and memory B cells during so-called germinal center (GC) reaction and cytokine production may also serve as correlates of vaccine-induced protection [9]. Thus, to indirectly estimate the vaccine-induced protection, it has been suggested to use multiple immune correlates/surrogates of protection [9].

In light of this, it is clear that it is of great importance to improve immune responses to influenza vaccines that correlate with protection. So far, much attention has been paid to the virus-related factors contributing to immune responses correlating with protection, while the influence of host-associated variables, such as genetic factors, biological sex, age, and hormonal and (micro)nutritional status-related variables present prior to vaccination have been poorly investigated [12]. This seems particularly important as increased dose of antigen does not proportionally increase GC output [13]. Accordingly, it is important to unveil targetable mechanisms of influenza-vaccine-induced protective immune responses, and subsequently to identify putative extrinsic factor/s that positively impact/condition these immune mechanisms even prior to vaccination, and thereby “optimize” immune responses to the vaccine. In this respect, this paper’s research was focused on the putative significance of supplementation with vitamins, oligoelements, and modified amino acids, such as N-acetyl cysteine (NAC), which up to now have been ordered and used predominantly to prevent viral infection and promote recovery from these infections [14,15,16,17,18,19,20,21]. This research is particularly justifiable as: (i) variety of observational and some interventional studies report that adequate micronutrient status or micronutrient supplementation is associated with enhanced vaccine responses [22], (ii) gaps in several micronutrients, including those incorporated into this supplementation, are reported [22], and (iii) such an intervention could be implemented safely and inexpensively. The influence of the above proposed antioxidant/immunomodulatory supplementation (started before vaccination) was attested for its capacity to optimize immune response to QIV. For this purpose, serum-virus-influenza-specific IgG antibody titre and putative GC B cell- and GC CD4+ T cell response-related correlates/surrogates of protection were evaluated following the primary QIV immunization, and the second QIV challenge (mimicking in some way subsequent natural virus influenza infection). This was particularly important as immune memory, a critical correlate of the protection against subsequent infections, was not quantified in response to the first virus vaccine challenge (QIV immunization) [9]. Specifically, following the first and second QIV challenge, apart from the serum total and the neutralizing-virus influenza-specific IgG titre (which are commonly used as the end-point immune correlates of protection), draining lymph nodes (dines) and spleens were investigated for the frequency of GC B lymphocytes and GC CD4+ (T helper, Th) lymphocytes. Additionally, the frequency of cells belonging to Tfh lymphocyte subsets, viz., T follicular helper (Tfh) cells, important for B cell differentiation into high-affinity antibody-secreting plasma cells and frequency memory B cells, and T follicular regulatory (Tfr) cells acting directly and indirectly (in a Tfh-dependent manner) on GC B cells to restrict their response, was determined [23,24]. Furthermore, Th lymphocytes were examined for synthesis of IL-21, the cytokine with a key role in GC biology through its independent actions on T and B cells [25]. Moreover, given that Th1 and Th2 cells have differential capabilities in stimulating B lymphocytes to secrete antibody isotypes with different functional properties (and thereby have a protective capacity) [26], the ratio of their production levels in dLN cell and splenocyte cultures, as well as the serum IgG2a/IgG1 titres ratio, were also determined. Of note, the evaluation of the aforementioned immune response parameters could also give a mechanistic insight into the action of antioxidants/immunomodulators used for supplementation. Thus, the ultimate goal of this study was to investigate (based on immune protection correlates/surrogates) if there is a scientific rationale for considering the use of such an antioxidant/immunomodulatory supplementation to include support to/optimization of immune responses to the influenza virus vaccine in the spectrum of indications for its use.

## 2. Materials and Methods

### 2.1. Experimental Animals

The study encompassed 8–10-week-old female mice of the BALB/c strain (6 per group). Mice were bred at the Institute of Virology, Vaccines and Sera “Torlak”, Belgrade, Serbia, and kept under the standard housing conditions (12/12-h cycle of light and dark, and a controlled temperature and humidity environment) with ad libitum access to food and water. The animal care and all experimental procedures were performed in accordance with the Directive 2010/63/EU of the European Parliament and of the Council on the Protection of Animals Used for Scientific Purposes, and in accordance with the governmental regulations (Law on Animal Welfare, “Official Gazette of Republic of Serbia”, No. 41/2009). The study protocol was evaluated by the Animal Care and Use Committee of the Institute of Virology, Vaccines and Sera “Torlak” and approved by the Ministry of Agriculture, Forestry and Water Economy of the Republic of Serbia—Veterinary Directorate (document number: 323-07-12928/2022-05).

### 2.2. Immunization and Treatment

**Figure 1:**
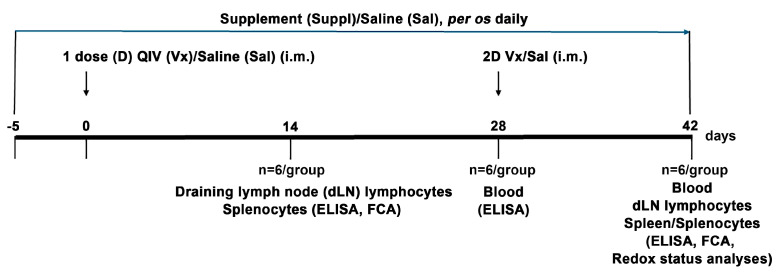
Immunization, treatment, blood/organs collection and analysis timeline. The mice were immunized intramuscularly with two doses (D) of QIV (Vx) or saline (Sal) on days 0 (1D) and 28 (2D). Per os daily treatment with antioxidants/immunomodulators in saline (Suppl) or administration of same volume of saline alone (Sal) started five days before the first injection of QIV or saline, and lasted until the day of sacrifice (mice were sacrificed 28 or 42 days after the first injection of QIV). Blood was collected 28 and 42 days after the first injection (14 days after the second injection) of QIV and analyzed using ELISA. Draining lymph nodes (dLNs) and spleens were collected 14 and 42 after the first injection (14 days after the second injection) of QIV, and dLN lymphocytes and splenocytes were separated and analyzed by flow cytometry. Redox status was analyzed in spleens on the 42nd day post-immunization (14 days after the second injection of QIV).

Experimental animals were immunized with QIV for the 2022/2023 season, which contained: A/Victoria/2570/2019 (H1N1)pdm09-like virus, A/Darwin/9/2021 (H3N2)-like virus, B/Austria/1359417/2021 (B/Victoria lineage)-like virus, and B/Phuket/3073/2013 (B/Yamagata lineage)-like virus (Sanofi Pasteur–Val de Reuil, Val de Reuil, France). The vaccine was applied intramuscularly, with 50 μL in each caudal thigh and in a dose containing 3 μg of virus surface hemagglutinin (HA) of each virus strain/lineage, the human-equivalent dose [25]. Immunization was carried out at two time points, with an interval of 28 days between them (Figure 1). Control mice were injected with saline in the same way. All mice were monitored daily by veterinarians and researchers. They showed no signs of local or systemic reactions.

Five days before the first injection, the immunized and control mice were randomly divided into two groups: one group was assigned to supplementation [3.78 mg/mouse/day, the human-equivalent dose [27] of antioxidants/immunomodulators in saline], whereas another group was administered with the same volume of saline. The supplement contained a combination of antioxidants/immunomodulators in the same ratio as in the commercially available combination for human use of antioxidants/immunomodulators BiVits^®^ ACTIVA Recovery (AbelaPharm, Belgrade, Serbia), viz., 500.0 mg vitamin C (L-ascorbic acid), 25.0 µg vitamin D3 (cholecalciferol), 10.0 mg zinc (zinc-citrate), 1.0 mg manganese (manganese gluconate), 27.5 µg selenium (L-selenomethionine), and 300.0 mg NAC obtained by the process of natural fermentation in saline and in the human-equivalent dose [27]. The supplement or saline alone was administered per os daily until the day of sacrifice (Figure 1).

### 2.3. Reagents and Antibodies

Ketamine (Ketamidor, Richter Pharma AG, Wels, Austria) and xylasine (Xylased, Bioveta, Ivanovice na Hané, Czech Republic) were used for making the anesthesia cocktail.

Goat anti-mouse IgG, IgG1, and IgG2a antibodies conjugated with horseradish peroxidase (HRP) (Jackson ImmunoResearch Laboratories Inc., WestGrove, PA, USA), and o-phenylenediamine (OPD) from Sigma were used for serum-antibody-titre determination.

The following antibodies: fluorescein isothiocyanate (FITC)-conjugated anti-B220 (CD45R, clone RA3-6B2), FITC-conjugated anti-FoxP3 (clone FJK-16s), and phycoerythrin (PE)-conjugated anti-CD95 (clone 15A7) were obtained from eBiolegend (Carlsbad, CA, USA); PE-conjugated anti-CD4 antibody (clone RM4-5) was purchased from Biolegend (San Diego, CA, USA); peridinin chlorophyll protein cyanine (PerCP-Cy™5.5)-conjugated anti-CD4 antibody (clone RM4-5), perCP-Cy™5.5- or PE-conjugated anti-mouse CXCR5 (Clone 2G8), and Alexa Fluor (AF) 647-conjugated anti-human/mouse Bcl6 (K112-91) were acquired from BD Biosciences Pharmingen (Mountain View, CA, USA) and were used for immunostaining and flow cytometric analysis (FCA). Additionally, rabbit polyclonal anti-IL-21 antibody (Merck KGaA, Darmstadt, Germany) and secondary FITC-conjugated goat anti-rabbit Ig antibody (BD Biosciences Pharmingen) were used. For analyses of cell proliferation, FITC- and PE-conjugated anti-Ki67 (clone SolA15; eBioscience Inc. San Diego, CA, USA) were used. All antibodies used in the experiments specifically react with mouse antigens and were tested for use in flow cytometry by the manufacturers. Target-cell-antigen specificity for all the antibodies used was also confirmed in our previous [28] and present studies, in single- or multi-color analyses, and using antigen-positive and -negative cell types.

The cells were cultivated in medium RPMI 1640 (Sigma-Aldrich Chemie GmbH, Taufkirchen, Germany), with 10% fetal calf serum (FCS) (Gibco, Grand Island, NY, USA), 2 mM L-glutamine (Serva, Heidelberg, Germany), 1 mM sodium pyruvate (Serva), 100 unit/mL penicillin (ICN, Costa Mesa, CA, USA), and 100 μg/mL streptomycin (ICN).

Bovine serum albumin (BSA) obtained from Fluka AG (Chemie) GmbH (Buchs SG, Switzerland) was used in ELISA for antibody titre determination.

Phorbol 12-myristate 13-acetate (PMA) and ionomycin, both purchased from Sigma-Aldrich Chemie GmbH and brefeldin A (eBioscience), were used to determine IL-21 expression.

Legend max™ mouse interferon (IFN)-γ and interleukin (IL)-4 ELISA kits (Biolegend) were used to determine cytokines in cell culture supernatants.

### 2.4. Serum Collection

Animals were deeply anesthetized with ketamine [80 mg/kg body weight (BW)]/xylazine (8 mg/kg BW) cocktail and bled from retroorbital sinuses. Sera were collected after centrifugation (2000× *g*, 10 min) of coagulated blood samples, decomplemented (56 °C, 30 min), and stored (−20 °C) for further analyses.

Blood samples were collected at two time points: 28 days after the injection of the first dose of QIV (when the titre reaches its maximum, according to a preliminary experiment) or saline, and 14 days after the second injection of QIV or saline (Figure 1).

### 2.5. Total IgG and IgG Subclass Titre Determination

The total IgG and IgG subclass titres (IgG1 and IgG2a) of antibodies against each virus strain were determined by end point dilution enzyme-linked immunosorbent assay (ELISA). As previously described [29], serial dilution of sera prepared in PBS with 1% BSA were added to Nunc MaxiSorp 96 well flat bottom plates pre-coated with 2.5 μg of HA/mL of QIV or inactivated H1N1 or H3N2 or B/Austria/1359417/2021 (B/Victoria lineage)-like or B/Phuket/3073/2013 (B/Yamagata lineage)-like viruses (incorporated in QIV), and incubated for one hour at RT, then washed three times with 0.05% Tween 20/PBS and once with PBS. IgG and IgG subclass-specific goat anti-mouse antibodies, conjugated with HRP, were added in appropriate dilutions: IgG (1:10,000), IgG1 (1:10,000), IgG2a (1:10,000) and incubated for one hour at RT. After washing, o-phenylenediamine with H_2_O_2_ was added and the reaction was stopped after 15 min with 2M H_2_SO_4_. Optical density was measured with Multiscan Ascent (Labsystems) at 490 nm and 620 nm. Titres were calculated as the reciprocal of the highest dilution of test sera that gave an absorbance reading value of 3 standard deviations above the control sera at an equivalent dilution.

### 2.6. Neutralizing Antibody Titre Determination

To determine neutralizing antibody titre, microneutralization assay was performed according to WHO protocol [29]. Firstly, 50% median tissue culture infectious dose (TCID50) for each virus strain from the vaccine was determined. Serially diluted receptor-destroying enzyme-treated sera were pre-incubated with a 100× TCID50 of live virus prior to the addition of Madin–Darby canine kidney (MDCK) cells. After overnight incubation, the cells were stained with crystal violet dye. The neutralization titre was expressed as the reciprocal of the highest dilution of serum that gave 50% neutralization of 100 × TCID50 of virus in MDCK cells.

### 2.7. Isolation of Cells from dLNs and Spleens

Cellular immune response was examined at two time points: two weeks after the first injection of QIV and two weeks after the second injection of QIV. Spleens and dLNs (inguinal) were removed and passed through a 60 μm sieve screen in 10% FCS/RPMI 1640 under sterile conditions. Splenic cell suspensions were subjected for 10 min to lysis buffer (0.15 M NH_4_Cl, 1mM KHCO_3_, and 0.1 mM Na_2_EDTA in H_2_O, pH 7.2) in order to remove red blood cells, followed by centrifugation (300× *g*, 10 min) and washing. Then, cells were resuspended in 10% FCS/PBS and counted in 0.2% trypan blue dye using the improved Neubauer haemocytometer. Isolated cells were examined for phenotype characteristics, cytokine production, and proliferation capability (Figure 1).

### 2.8. Cell Culturing and Stimulation

Splenic and dLN cell suspensions were adjusted to 5 × 10^5^ cells/well, and cells were cultivated in medium (10% FCS/RPMI 1640) in Nunc MaxiSorp 96 well U-bottom plates at 37 °C in a 5% CO_2_ humidified air atmosphere. After 72 h of (re)stimulation with QIV (5 μg of HA/mL), cells were subjected to immunostaining and FCA for assessment of proliferative cell response and IL-21 expression. Additionally, the supernatants were collected and stored at −20 °C until assessment of IFN-γ and IL-4 production by ELISA.

### 2.9. Cell Proliferation Assessment

Following in vitro (re)stimulation, the proliferation capacity of CD4+CXCR5+ T cells and B220+CD95+ B cells from spleen and dLNs were examined. Briefly, after surface immunostaining of CD4 and CXCR5 or B220 and CD95 antigens, cells were fixed/permeabilized overnight at 4 °C and then subjected to intracellular staining with anti-Ki67 and subsequently to FCA.

### 2.10. Intracellular Cytokine Production Assessment

The production of IL-21 was analyzed in freshly isolated and QIV HA-restimulated cultured splenocytes and dLN cells. Before IL-21 immunostaining, cells were activated with 200 ng/mL PMA and 400 ng/mL ionomycin in a 5% CO_2_ humidified atmosphere at 37 °C for 4 h. In addition, 3 μg/mL of brefeldin A was added. 

### 2.11. Immunostaining and FCA

#### 2.11.1. Surface Antigens

The cells were dispersed into BD tubes (5 × 10^5^ cells), washed in 2% FCS/0.1% NaN_3_/PBS, and then incubated with monoclonal antibodies against surface antigens (CD4, CXCR5, B220, CD95) for 30 min at 4 °C. After incubation, cells were washed and resuspended in 0.1% NaN_3_/PBS for FCA or fixed and permeabilized overnight at 4 °C for intracellular staining using fixation/permeabilization buffers (eBioscience) according to the manufacturer’s instructions.

#### 2.11.2. Intracellular Antigen

The activated cells immunolabeled for surface antigens were subjected to overnight fixation/permeabilization, and then washed and incubated with fluorochrome-conjugated or unconjugated antibodies specific to intracellular antigens (FoxP3, Bcl6, Ki67, IL-21) for 30 min at RT. The cells immunostained with unconjugated anti-IL-21 antibody were washed and subjected to FITC-conjugated goat anti-rabbit Ig for a further 30 min.

After PBS washing, the cells immunolabeled for intracellular antigens were acquired on a FACSVerse flow cytometer (Becton Dickinson, Mountain View, CA, USA). 

#### 2.11.3. FCA

To secure the relevant number of events for statistical analysis in each gate, 100,000 events per sample were acquired on a flow cytometer (FACSVerse) as calculated by Poisson statistics. Unstained cells, fluorochrome-matched isotype staining, and secondary antibody-only controls were used for the optimization of analyses. With the exception of markers exhibiting clear bimodal staining [30], fluorescence minus one (FMO) controls were used to determine the gating boundaries. The frequency of marker-positive cells was determined using FlowJo software version 7.8. (TreeStar Inc., Ashland, OR, USA).

### 2.12. ELISA

Concentrations of IFN-γ and IL-4 were determined in supernatants from splenocyte and dLN cell cultures using Biolegend ELISA kits following the manufacturer’s instructions. Briefly, after washing the pre-coated plates, assay buffer and appropriately diluted standards or samples (50 μL/well) were added to the wells and incubated for two hours at RT in a shaker. After the washing step, detection antibody was added and incubated for an additional hour (RT on shaker). Following washing, avidin-HRP solution was added and incubation continued for 30 min at RT while shaking. After washing, the substrate solution was added and incubated for the following 15 min in the dark. The reaction was stopped with the stop solution and OD was measured with Multiscan Ascent at 450 nm and 570 nm. The standard curve to determine concentrations was calculated for each assay with the limit of detection of 0.5 pg/mL and 8 pg/mL for IL-4 and IFN-γ, respectively.

### 2.13. Redox Status Parameters Assessment

Frozen samples of spleens extirpated two weeks after the second injection of QIV were homogenized in 0.1 M phosphate buffer (pH = 7.4) in a 1:9 weight-to-volume ratio using the Tehtnica homogenizer (Železniki, Slovenia) and then centrifuged for 10 min at 800× *g*, and then for 20 min at 9500× *g* to obtain supernatants. All the steps were carried out at 0–4 °C. In the supernatants, redox parameters were assessed. The obtained values were normalized to the protein concentration of supernatants, which was determined using the Bradford method.

The level of superoxide anion radical O2•− was estimated according to the rate of yellow nitroblue tetrazolium reduction to blue diformazan [31].

Total pro-oxidant capacity (TOC) was determined using a slightly modified spectrophotometric method introduced by Erel [32,33]. The method is based on the ability of oxidants such as hydrogen peroxide and lipid hydroperoxide to oxidize the ferrous ion-o-dianisidine complex to ferric ion, which in reaction with xylenol orange in an acidic medium makes a colored complex. The intensity of color is proportional to the total amount of oxidant molecules in the sample. The assay is calibrated with hydrogen peroxide in concentration ranging from 10 to 200 μmol/L.

The level of advanced oxidation protein products (AOPP) was estimated by measuring the absorbance at 340 nm of complex formed in their reaction with glacial acetic acid potassium-iodide [34]. Chloramine T, a compound exhibiting specific absorption maximum at 340 nm, was used as a standard with a concentration range of 10 to 100 μmol/L.

The activity of Cu/Zn superoxide dismutase (SOD) was measured according to the slightly modified method of Misra and Fridovich [35]. This method is based on the ability of SOD to inhibit auto-oxidation of epinephrine in alkaline medium. The enzyme activity is calculated as the percentage of inhibition of epinephrine auto-oxidation.

The level of SHG was determined by a modified Ellman’s method [33], using 10 mmol/L dinitrodithiobenzoic acid as a reagent. This reagent reacts with aliphatic thiol compounds in a basic environment (pH 9.0) and this reaction generates 1 mol p-nitrophenol anion per mol of thiol. The absorbance was measured at 412 nm. The calibration of the method was achieved with the reduced glutathione in the concentration range from 0.1 to 1.0 mmol/L.

The level of glutathione (GSH) was determined based on its reaction with 5,5′-dithiobis-2-nitrobenzoic acid, leading to the formation of a yellow complex 5-thio-2-nitrobenzoate, which is measured after the deproteinization with sulfosalicylic acid at 412 nm [36]. Reduced glutathione in a range from 0.5 to 2 μmol/L was used for the calibration.

The levels of O2•−, TOC, and SOD activity were measured on the ILAB 300 Plus analyzer (Instrumentation Laboratory, Italy), whereas AOPP, SHG, GSH, and protein concentrations were measured on a continuous spectrophotometer (Pharmacia LKB, UK).

### 2.14. Statistics

The assessment of statistically significant differences between the groups was done by the Kruskal–Wallis test followed by the Mann–Whitney U test. All statistical analyses were performed using GraphPad Prism 9 software (GraphPad Software, Inc., La Jolla, CA, USA). Data are presented as median ± interquartile range, unless otherwise specified. Differences are considered significant when *p* ≤ 0.05.

## 3. Results

### 3.1. Antioxidant/Immunomodulatory Supplementation Increased the Total Titre of Influenza Virus-Specific IgG in Sera and the Frequency of GC B Cells in dLNs and Spleens of Mice Injected with one Dose of QIV

**Figure 2:**
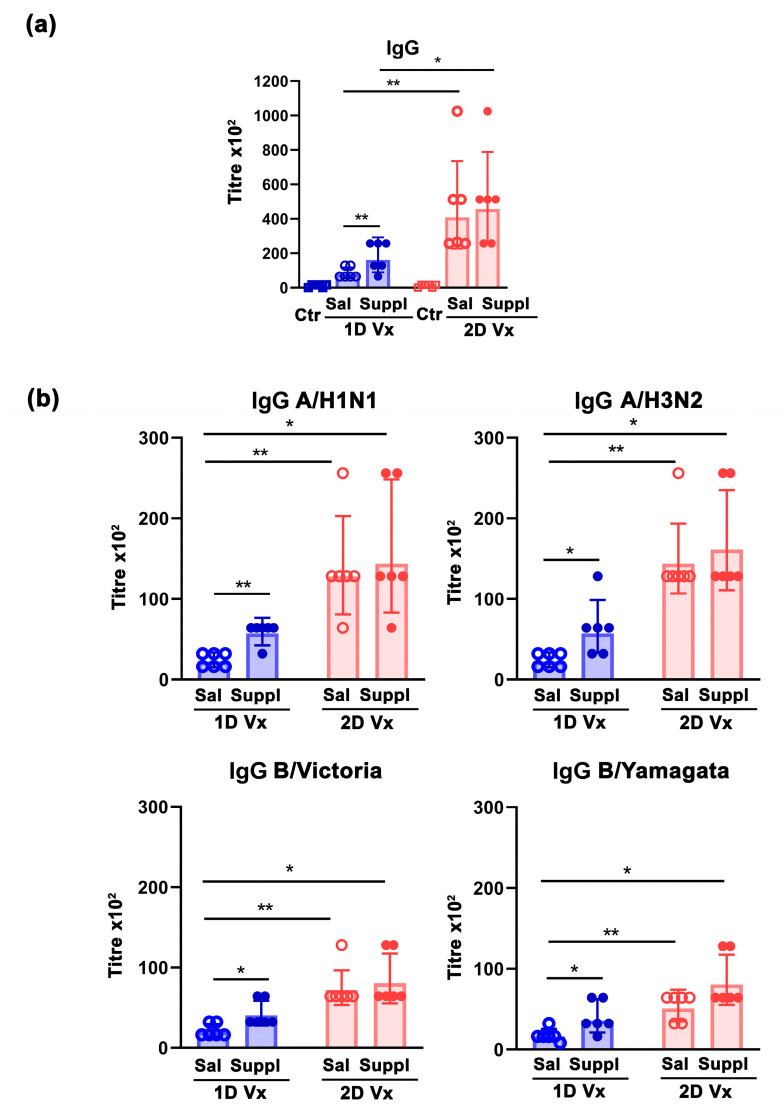
Supplementation with antioxidants/immunomodulators increased influenza-specific IgG response in mice injected with one dose of QIV. Graph bars indicate serum geometric mean titres of the total (**a**) QIV antigen-specific IgG antibodies and (**b**) IgG antibodies specific to influenza subtypes: A/H1N1, A/H3N2, B/Victoria lineage, and B/Yamagata lineage in mice injected with one dose (1D) and two doses (2D) of QIV (Vx) and treated with antioxidant/immunomodulatory supplement (Suppl) or saline (Sal), and in mice injected intramuscularly with Sal and treated per os with Suppl (control group, Ctr). Titres were determined 28 days after the first injection of QIV/Sal (1D) and 14 days after the second injection of QIV/Sal (2D). Error bars indicate the 95% confidence interval (CI). n = 6 mice/group. * *p* ≤ 0.05, ** *p* ≤ 0.01.

Given that, in contrast to QIV-immunized mice, the geo means of the total serum titre of influenza virus-specific IgG in mice that received one or two injections of saline and were supplemented with antioxidants/immunomodulators (Controls) were equal to zero, it is obvious that all mice that received QIV developed influenza virus-specific antibody response (Figure 2a). In mice injected with one dose of QIV, on the 28th day post-immunization, the total serum titre of influenza virus-specific IgG was higher (*p* ≤ 0.05) in supplemented animals than in those administered with saline (Figure 2a). As expected [37], the second injection of QIV boosted the total serum titre of influenza virus-specific IgG, so 14 days after the injection it was dramatically higher (*p* ≤ 0.01) compared with that measured on the day when it was administered (Figure 2a). The supplementation did not significantly influence this response (Figure 2a).

Considering that antibodies should have sufficient breadth to affect all virus strains [9], serum titres of IgG antibodies against each of the four virus influenza strains from QIV were measured (Figure 2b). It is noteworthy that, following immunization, and in response to the supplementation, similar patterns of changes to those described for the serum total QIV antigen-specific IgG titres were observed for the serum titres of IgG against each of the four influenza strains from the vaccine (Figure 2b).

**Figure 3:**
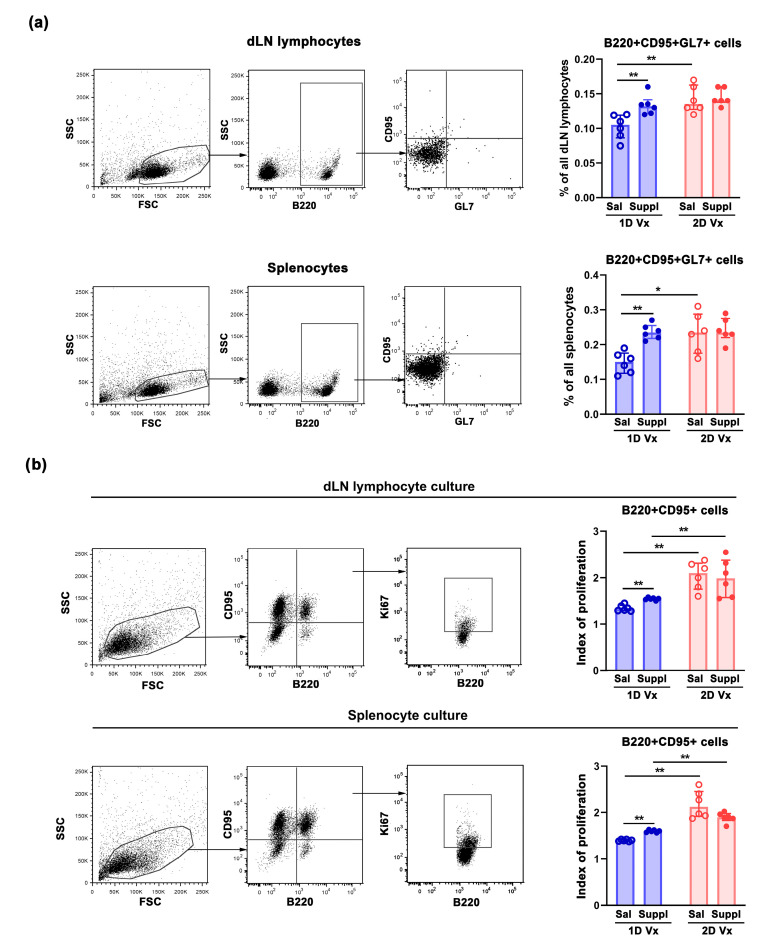
Supplementation with antioxidants/immunomodulators increased the frequency of GC B cells in dLNs and spleens, and their proliferative response to QIV antigens in vitro. (**a**) Graph bars display the percentages of B220+CD95+GL7+ cells (GC B cells) among (upper row) draining lymph nodes (dLN) lymphocytes and (lower row) splenocytes from mice 14 days after one dose (1D) and two doses (2D) of QIV (Vx)) in mice treated with antioxidant/immunomodulatory supplement (Suppl) or saline (Sal). Flow cytometry dot plots indicate the gating strategy for flow cytometry analyses of GC B cells among (upper row) dLN lymphocytes and (lower row) splenocytes. Flow cytometry dot plots indicate (right) GL7 vs. CD95 staining of B220+ cells gated as shown in middle flow cytometry dot plots from (left) “lymphocyte” gate. (**b**) The graph bars indicate proliferative index of GC B cells, i.e., the increase in the frequency of proliferating Ki67+ cells among B220+CD95+ cells in the presence of QIV antigens over that in their absence in cultures of (upper row) dLN lymphocytes and (lower row) splenocytes from mice injected with 1D and 2D of QIV (Vx) and administered with Suppl or Sal. Flow cytometry dot plots indicate the gating strategy for flow cytometry analysis of the frequency of Ki67+ cells among (upper row) B220+CD95+ dLN lymphocyte and (lower row) splenocyte cultures. Flow cytometry dot plots indicate (right) Ki67 vs. CD95 staining of B220+ cells gated as shown middle dot plots. B220+ cells were gated within “lymphocytes” as shown in left flow cytometry dot plots. Data are presented as median and interquartile range (IQR) (individual data points are incorporated into bars). n = 6 mice/group. * *p* ≤ 0.05, ** *p* ≤ 0.01.

In accordance with IgG antibody response to the first injection of QIV, the frequency of B220+CD95+GL7+ cells, presumably GC B cells [38], in dLNs and spleens from supplemented mice was higher (*p* ≤ 0.01) compared to mice administered with saline (Figure 3a). The second injection of QIV additionally increased the frequency of GC B cells in secondary lymphoid organs (SLOs) (*p* ≤ 0.01), so 14 days after the second injection their frequency in dLNs (*p* ≤ 0.01) and spleens (*p* ≤ 0.05) was higher than at the time when the second injection was applied (Figure 3a). The treatment with the supplement did not statistically significantly influence the frequency of B220+CD95+GL7+ cells either in dLNs or spleens from mice administered with two injections of QIV (Figure 3a).

Next, to enlighten the putative mechanisms underlying the increase in the frequency of B cells participating in GC reaction (GC B cells), their proliferative index in response to restimulation with QIV antigens (viz., the increase in the frequency of proliferating Ki67+ cells in the presence of QIV antigens over that in their absence) in vitro was investigated (Figure 3b). GC B cells were defined by surface co-expression of B220 and the pro-apoptotic receptor CD95 (also known as APO-1/Fas), the crucial regulator of B-cell–T-cell interactions in SLOs [38,39]. The proliferative index of GC B cells from cultures of dLN lymphocytes and splenocytes from mice injected with one dose of QIV and treated with the supplement was greater (*p* ≤ 0.01) than when compared with the proliferative index of corresponding cells from mice injected with one dose of QIV and administered with saline (Figure 3b). As expected from previous findings, GC B cells from dLNs and spleens from mice injected with two doses of QIV and administered with saline exhibited a greater (*p* ≤ 0.01) proliferative index compared with mice injected with one dose of QIV and administered with saline (Figure 3b). Furthermore, the supplementation with antioxidants/immunomodulators did not statistically significantly influence the proliferative response of GC B cells in cultures from either dLNs or spleens of mice receiving two doses of QIV (Figure 3b).

### 3.2. Supplementation with Anti-Oxidants/Immunomodulators Increased the Frequency of Tfh Cells Only in SLOs from Mice Injected with One Dose of QIV

**Figure 4:**
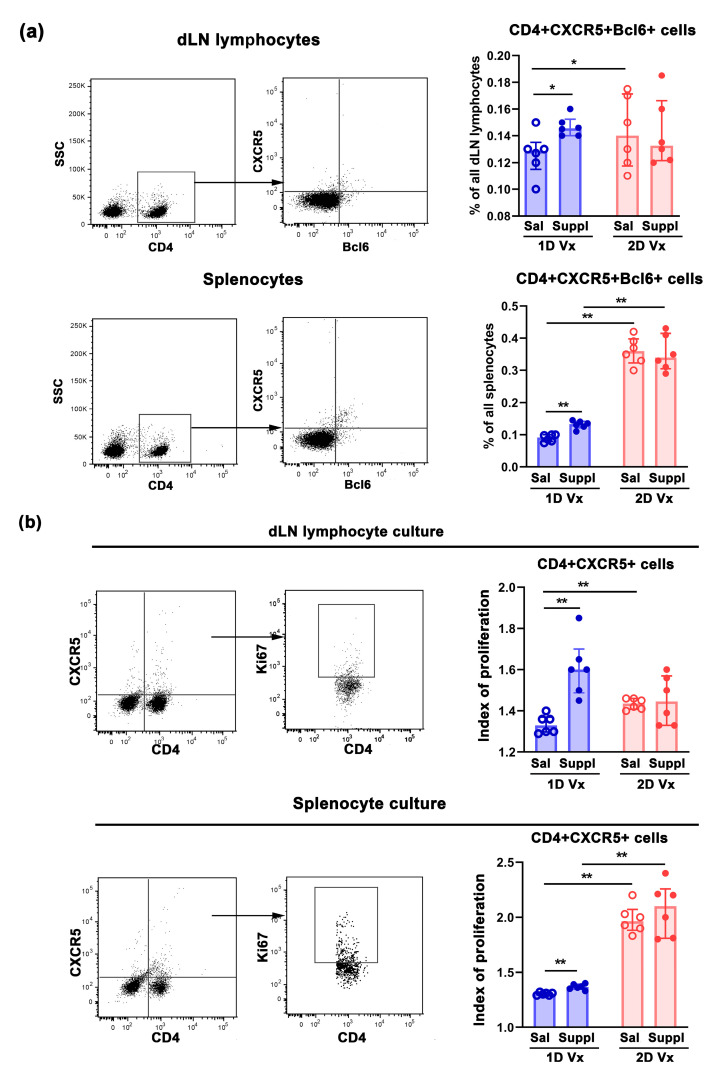
The supplementation with antioxidants/immunomodulators increased the frequency of GC CD4+ T cells in dLNs and spleens from mice injected with one dose of QIV. (**a**) The graph bars display the percentages of GC CD4+CXCR5+Bcl6+ T cells among (upper row) draining lymph node (dLN) lymphocytes and (lower row) splenocytes from mice injected with one dose (1D) and two doses (2D) of QIV (Vx), and administered with saline (Sal) or supplement (Suppl) determined by flow cytometry. The analyses were performed 14 days after injection of the first and second doses of QIV. The flow cytometry dot plots indicate the gating strategy for GC CD4+CXCR5+Bcl6+ T cells among (upper row) dLN lymphocytes and (lower row) splenocytes. (Right) CXCR5 vs. Bcl6 staining of CD4+ cells gated as indicated in (left) flow cytometry dot plot. CD4+ cells were gated within lymphocyte gates shown in Figure 3a. (**b**) The graph bars display the proliferative index of GC CD4+CXCR5+ T cells, i.e., the increase in frequency of proliferating Ki67+ cells among GC CD4+CXCR5+ T cells in the presence of QIV antigens over that in their absence in cultures of (upper row) dLN lymphocytes and (lower row) splenocytes from mice injected with 1D and 2D of QIV (Vx) and administered with Supp or Sal in response to restimulation with QIV antigens. Flow cytometry dot plots indicate Ki67+ staining of CD4+ CXCR5+ (upper row) dLN lymphocytes and (lower row) splenocytes from lymphocyte gates shown in Fig 3b. Data are presented as median and interquartile range (IQR) (individual data points are incorporated into bars). n = 6 mice/group. * *p* ≤ 0.05, ** *p* ≤ 0.01.

Considering that the initiation of the GC requires both CD4+ T cells and B cells to be activated by cognate antigen [40], the frequency of GC T cells (defined by the surface expression of CXCR5 and Bcl6 molecules) [41] was investigated in SLOs of mice immunized with QIV. The frequency of CD4+CXCR5+Bcl6+ cells increased in dLNs (*p* ≤ 0.05) and spleens (*p* ≤ 0.01) of supplemented mice administered with one dose of QIV compared with those administered with one injection of QIV and saline (Figure 4a). In mice administered with saline, their frequency in dLNs (*p* ≤ 0.05) and spleens (*p* ≤ 0.01) was higher than in mice injected with two doses of QIV and in respect to those administered with one dose of the vaccine (Figure 4a). The supplementation did not influence the frequency of CD4+CXCR5+Bcl6+ cells in either dLNs or spleens from mice administered with two injections of QIV (Figure 4a). In mice supplemented with antioxidants/immunomodulators, their frequency was higher (*p* ≤ 0.01) in spleens, but not in dLN of animals administered with two injections of QIV, compared with those administered with one injection of QIV (Figure 4a).

To confirm the significance of QIV antigen-specific expansion of GC CD4+ T cells for the changes in their frequency, upon in vitro restimulation with QIV, their proliferative index (viz., the increase in the frequency of proliferating Ki67+ cells in the presence of QIV antigens over that in their absence) was calculated. Indeed, GC CD4+ cells from cultures of dLN lymphocytes and splenocytes of mice injected with one dose of QIV and supplemented with antioxidants/immunomodulators exhibited a greater (*p* ≤ 0.01) proliferative index when compared with cultures of mice injected with one dose of QIV and administered with saline (Figure 4b). Besides, the proliferative index of GC CD4+ cells was greater (*p* ≤ 0.01) in dLN lymphocyte and splenocyte cultures from mice administered with saline and injected with two doses of QIV compared with cultures from mice from saline-administered mice injected with one dose of the vaccine (Figure 4b). Of note, GC CD4+ cells from cultures of dLN lymphocytes and splenocytes of mice injected with two doses of QIV and supplemented with antioxidants/immunomodulators, and mice injected with two doses of the vaccine and administered with saline, exhibited a similar proliferative index (Figure 4b). Their proliferative index in splenocyte cultures from mice injected with two doses of QIV and administered with antioxidants/immunomodulators was greater (*p* ≤ 0.01) than in splenocyte cultures from mice supplemented with antioxidants/immunomodulators and injected with one dose of QIV (Figure 4b). However, in dLN cultures from mice supplemented with antioxidants/immunomodulators, the GC CD4+ cell proliferative index was similar in cultures from mice administered with one dose and two doses of QIV (Figure 4b).

**Figure 5:**
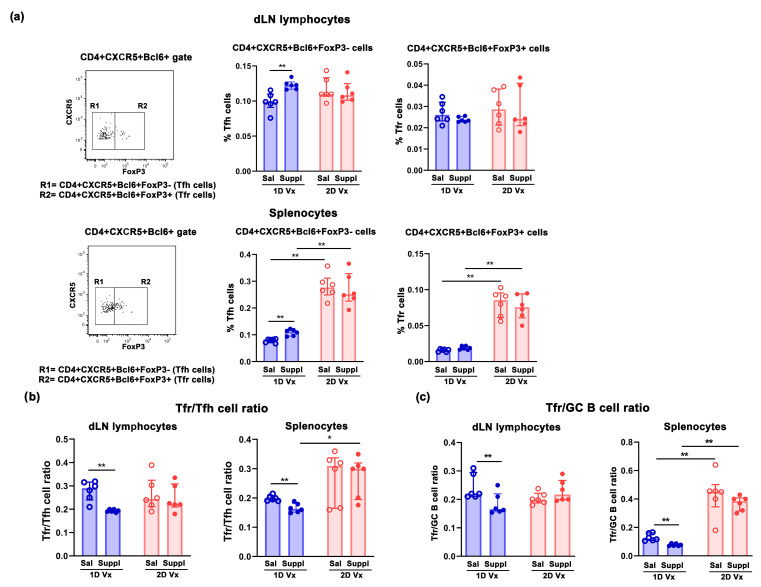
Supplementation with antioxidants/immunomodulators increased the frequency of Tfh cells and affected Tfr/Tfh and Tfr/GC B cell ratios in dLNs and spleens from mice injected with one dose of QIV. (**a**) Bar graphs indicate the frequency of CD4+CXCR5+Bcl6+FoxP3- (Tfh) cells and CD4+CXCR5+Bcl6+FoxP3+ (Tfr) cells among (upper row) draining lymph node (dLN) lymphocytes and (lower row) splenocytes from mice injected with one dose (1D) and two doses (2D) of QIV (Vx), and treated with saline (Sal) or supplement (Suppl). The analyses were performed 14 days after the first and second QIV injections. Flow cytometry dot plots indicate FoxP3 staining (R1 = FoxP3- cells; R2 = FoxP3+ cells) of CD4+CXCR5+ Bcl6+ (upper row) dLN lymphocytes and (lower row) splenocytes gated as shown in Figure 4a. Bar graphs indicate (**b**) Tfr/Tfh cell ratio, (**c**) Tfr/GC B cell ratio in dLN lymphocytes and splenocytes from mice injected with 1D and 2D of QIV (Vx) and administered with saline (Sal) or supplement (Suppl). Data are presented as median and interquartile range (IQR) (individual data points are incorporated into bars). n = 6 mice/group. * *p* ≤ 0.05, ** *p* ≤ 0.01.

Considering that CD4+CXCR5+Bcl-6+ T cells consist of Tfh cells and Tfr cells [41], dLN lymphocytes and splenocytes were simultaneously immunolabeled for CD4, CXCR5, Bcl6 and FoxP3 to delineate CD4+CXCR5+Bcl6+FoxP3- Tfh cells with a critical role in promoting GC B cell proliferation [42,43]. The supplementation with antioxidants/immunomodulators increased (*p* ≤ 0.01) the frequency of Tfh cells in dLNs and spleens of mice injected with one dose of QIV (Figure 5a). In mice administered with saline, 14 days after the second injection of QIV the frequency of Tfh cells was higher in dLNs and spleens compared with their frequency 14 days after the first injection, but this increase reached statistical significance (*p* ≤ 0.01) only in spleens (Figure 5a). The supplementation with antioxidants/immunomodulators did not statistically significantly influence their frequency in either dLNs or spleens from mice injected with two doses of QIV (Figure 5a). However, it is noteworthy that in mice supplemented with antioxidants/immunomodulators, the frequency of Tfh cells was higher (*p* ≤ 0.01) in spleens from mice administered with two injections of QIV compared with those administered with one injection of the vaccine (Figure 5a).

In addition, the frequency of CD4+CXCR5+Bcl6+FoxP3+ Tfr cells shown to control Tfh cell response and the production of IL-21 [23,44] which, in turn, regulates their development and function [45], was also determined. The supplementation with antioxidants/immunomodulators did not influence their frequency in SLOs of mice injected with either one or two doses of QIV (Figure 5a). In mice administered with saline and two injections of QIV, the frequency of Tfr cells was higher in dLNs and spleens compared with their counterparts administered with one injection of the vaccine, but this increase reached statistical significance (*p* ≤ 0.01) only in spleens (Figure 5a). Thus, in mice supplemented with antioxidants/immunomodulators their frequency was higher (*p* ≤ 0.01) among splenocytes from animals injected with two doses of QIV or compared with those injected with one dose of the vaccine (Figure 5a).

Next, given that Tfr/Tfh cell ratio is suggested to be more important for maintaining Tfh cell proliferation and function than the frequency of Tfr alone [23,46], this ratio was also investigated. The supplementation with antioxidants/immunomodulators shifted (*p* ≤ 0.01) Tfr/Tfh cell ratio towards Tfh cell in SLOs from mice immunized with one injection of QIV (Figure 5b). Administration of the second dose of QIV diminished (*p* ≤ 0.01) Tfr/Tfh dLN cell and splenocyte ratio, suggesting the formation of a new balance between these cells 14 days after the second injection of the vaccine (Figure 5b). Of note, in mice supplemented with antioxidants/immunomodulators, Tfr/Tfh splenocyte ratio was greater (*p* ≤ 0.05) in mice injected with two doses of QIV than in those injected with one dose of the vaccine (Figure 5b).

Considering that Tfr cells are suggested to exert direct suppressive effects on B cell differentiation [47], Tfr/GC B cell ratio was also determined (Figure 5c). The supplementation with antioxidants/immunomodulators in dLNs and spleens of mice injected with one dose of QIV shifted (*p* ≤ 0.01) this ratio towards GC B cells (Figure 5c). In spleens from mice administered with saline and injected with two doses of QIV Tfr/GC B cell ratio was greater (*p* ≤ 0.01) than in those injected with one dose of the vaccine (Figure 5c). This suggested a new balance between these two cell subpopulations following the second QIV injection in mice administered with saline (Figure 5c). The supplementation with antioxidants/immunomodulators did not influence Tfr/GC B cell ratio in SLOs (Figure 5c). Thus, this ratio was greater (*p* ≤ 0.01) in spleens from mice supplemented with antioxidants/immunomodulators and injected with two doses of QIV compared with those supplemented with antioxidants/immunomodulators and injected with one dose of the vaccine (Figure 5c).

### 3.3. Different Effects of Supplementation with Antioxidants/Immunomodulators on the Frequency of IL-21+CD4+ Cells in SLOs from Mice Injected with One and Two Doses of QIV

**Figure 6:**
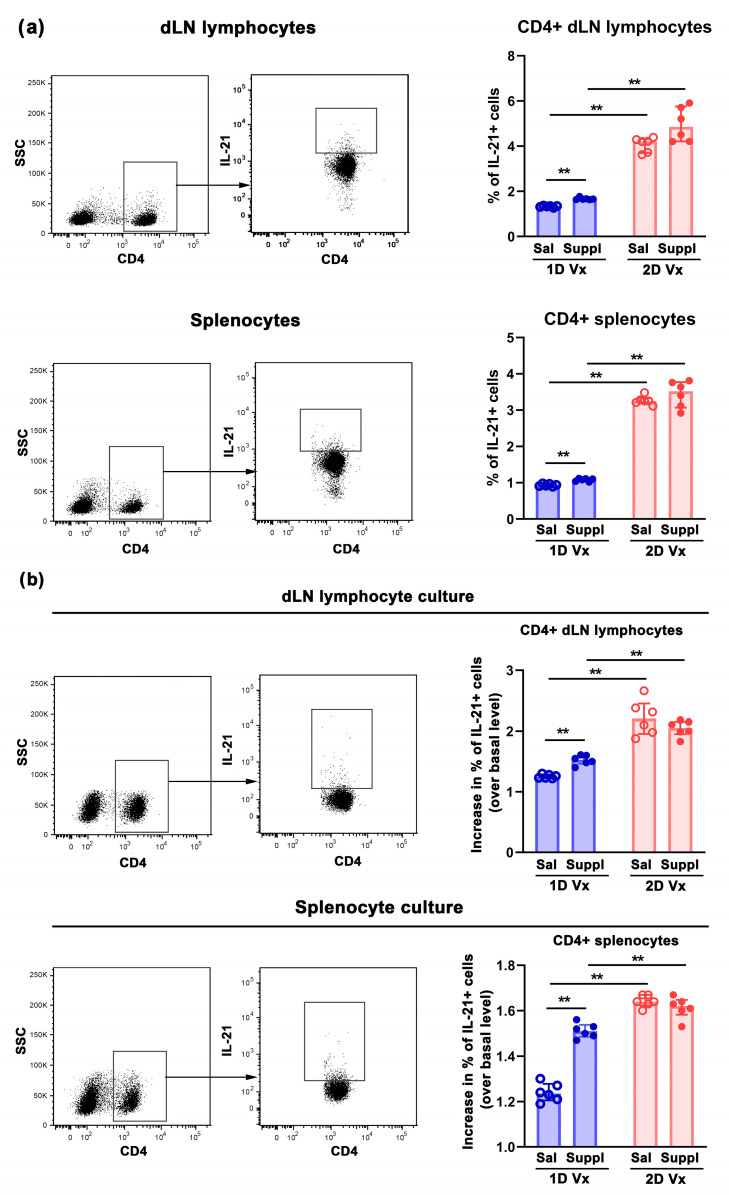
The supplementation with antioxidants/immunomodulators increased the frequency of IL-21+ cells among CD4+ cells from dLNs and spleens of mice injected with one dose of QIV. (**a**) The bar graphs display the percentage of IL-21+ cells among freshly isolated CD4+ (upper row) draining lymph node (dLN) lymphocytes and (lower row) splenocytes from mice injected with one dose (1D) and two doses (2D) of QIV (Vx), and administered with saline (Sal) or supplement (Suppl) 14 days after the first and the second doses of QIV. Flow cytometry dot plots indicate the gating strategy for flow cytometry analysis of IL-21+ cells among CD4+ cells in freshly isolated (upper row) dLN lymphocytes and (lower row) splenocytes from lymphocytes gated as shown in Figure 3a. (**b**) The bar graphs indicate the percentage of increase in the frequency of IL-21+ cells among CD4+ lymphocytes in QIV-supplemented over QIV-free (upper row) dLN lymphocyte and (lower row) splenocyte cultures from mice injected with 1D and 2D of QIV (Vx), and administered with Sal or Suppl. Flow cytometry dot plots indicate IL-21 staining of CD4+ cells from lymphocytes gated as presented in Fig 3b. Data are presented as median and interquartile range (IQR) (individual data points are incorporated into bars). n = 6 mice/group. ** *p* ≤ 0.01.

Considering that during CD4+ T cell-B cell collaboration, and apart from an essential role in B-cell activation, expansion, and plasma cell generation [48,49], IL-21 also plays an important role in Tfh cell differentiation and proliferation [50,51], its expression in CD4+ dLN lymphocytes and splenocytes was examined. The supplementation with antioxidants/immunomodulators increased (*p* ≤ 0.01) the frequency of IL-21+ cells among CD4+ cells from SLOs (Figure 6a). In mice administered with saline, fourteen days after the second QIV injection the frequency of IL-21+ cells among CD4+ cells from SLOs increased (*p* ≤ 0.01), compared with their frequency 14 days following the first injection of the vaccine (Figure 6a). In mice administered with two injections of QIV, their frequency was comparable in SLOs from mice who received the supplement and those administered with saline (Figure 6a). However, in mice supplemented with antioxidants/immunomodulators, the frequency of IL-21+ cells among CD4+ SLO cells was higher (*p* ≤ 0.01) in animals injected with two doses of QIV compared with those injected with one dose of the vaccine (Figure 6a). 

To confirm that the changes in the frequency of IL-21+ cells among CD4+ lymphocytes in SLOs were related to QIV immunization, their frequency was investigated in dLN lymphocyte and splenocyte cultures in the presence of QIV antigens, and in their absence and subsequently the fold increase was calculated. Indeed, in cultures of SLO cells from mice injected with one dose of QIV and supplemented with antioxidants/immunomodulators, the increase in the frequency of IL-21+ cells upon restimulation with QIV (over that in the absence of QIV) was greater (*p* ≤ 0.01) than in cultures from mice injected with one dose of QIV and administered with saline (Figure 6b). The increase in their frequency upon restimulation with QIV was also greater (*p* ≤ 0.01) in the culture of SLO cells from mice immunized with two doses of QIV and supplemented with saline, compared with those administered with saline and only one dose of the vaccine (Figure 6b). However, the increase in the frequency of IL-21+ cells upon restimulation with QIV was comparable in cultures of SLO cells from mice injected with two doses of QIV and supplemented with antioxidants/immunomodulators and in those from mice injected with two doses of the vaccine and administered with saline (Figure 6b). It is also noteworthy that this increase was more prominent (*p* ≤ 0.01) in cultures from mice immunized with two doses of QIV than in the corresponding cultures from mice immunized with only one dose of the vaccine (Figure 6b).

### 3.4. Antioxidant/Immunomodulatory Supplementation Affected IgG Subclass Profile Only in Mice Injected with Two Doses of QIV

**Figure 7:**
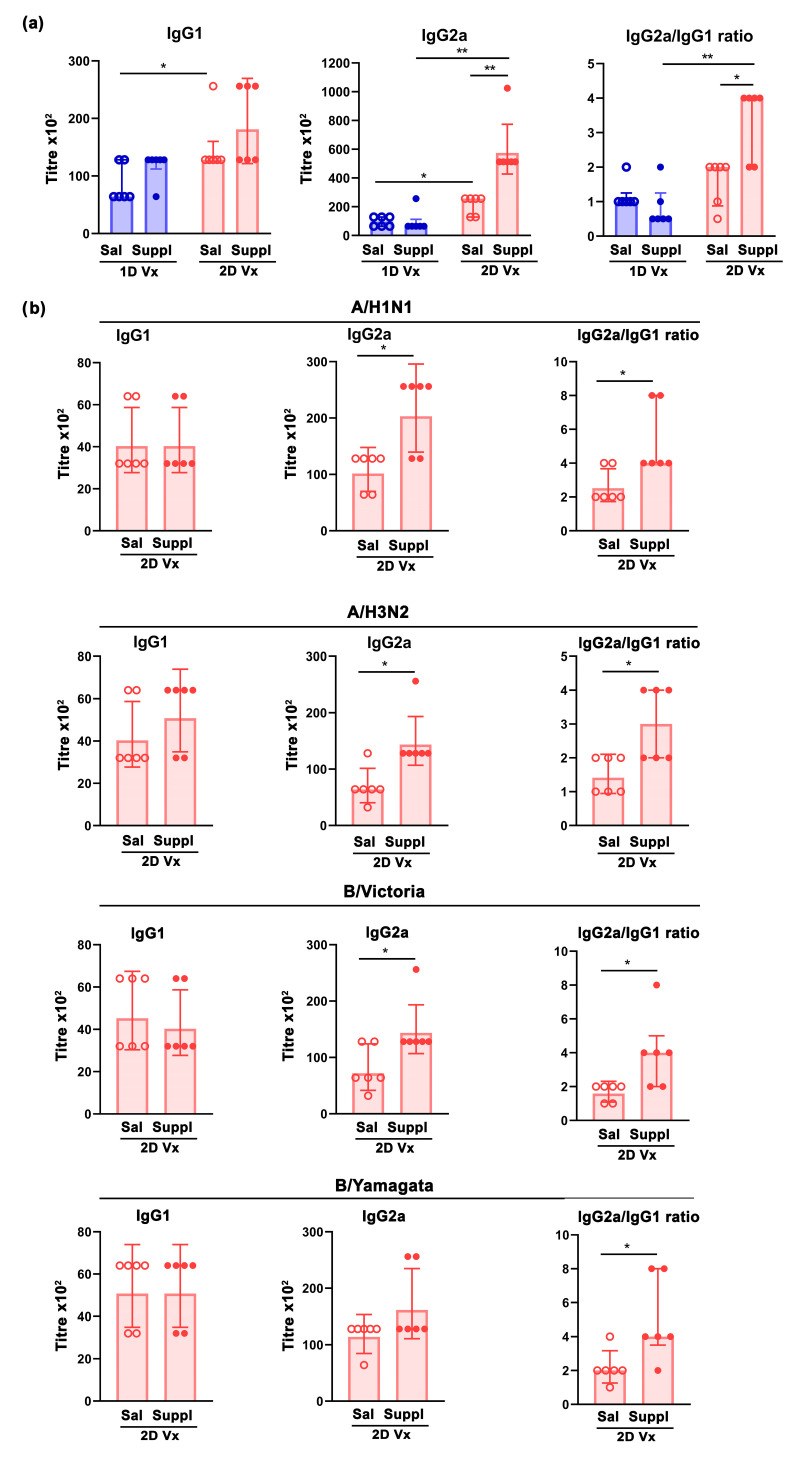
Supplementation with antioxidants/immunomodulators shifted serum IgG2a/IgG1 titre ratio towards IgG2a in mice injected with two doses of QIV. (**a**) Serum geometric mean titres of QIV antigen-specific IgG1 and IgG2a, and IgG2a/IgG1 ratio in mice injected with one dose (1D) and two doses (2D) of QIV (Vx) and administered with saline (Sal) or supplement (Suppl) determined 28 days after the first QIV dose and 14 days after the second QIV dose. (**b**) Serum geometric mean titres of IgG1 and IgG2a antibodies specific to influenza subtypes: A/H1N1, A/H3N2, B/Victoria lineage, and B/Yamagata lineage in mice injected with 2D of QIV (Vx) and administered with Sal or Suppl determined 14 days after the second QIV dose. Error bars indicate the 95% CI (n = 6 mice/group). * *p* ≤ 0.05, ** *p* ≤ 0.01.

Considering that CD4+ T cells providing help to B cells, apart from controling B-cell proliferation and differentiation into plasma cells, directly shape isotype switching [52], IgG subclass profile was examined. The supplementation with antioxidants/immunomodulators did not influence the serum titre of QIV antigen-specific IgG1 response in mice injected with either one or two doses of QIV (Figure 7a). However, the supplementation with antioxidants/immunomodulators increased (*p* ≤ 0.01) the titre of QIV antigen-specific IgG2a antibodies in mice administered with two doses of QIV (Figure 7a). In mice administered with saline, the serum titres of QIV antigen-specific IgG1 and IgG2a antibody were greater (*p* ≤ 0.05) in mice injected with two doses of QIV than in their counterparts injected with one dose of the vaccine (Figure 7a). Accordingly, in mice supplemented with antioxidants/immunomodulators, the serum titre of QIV antigen-specific IgG2a antibodies was greater (*p* ≤ 0.01) in animals injected with two doses of QIV than in their counterparts injected with one dose of QIV (Figure 7a). Consequently, unlike for mice injected with one dose of QIV (in whom the IgG2a/IgG1 response ratio did not differ between mice administered with saline and supplemented with antioxidants/immunomodulators), in mice injected with two doses of the vaccine IgG2a/IgG1 the response ratio was shifted (*p* ≤ 0.05) to the IgG2a response side in supplemented mice compared with those administered with saline (Figure 7a). In addition, in supplemented mice this ratio was shifted (*p* ≤ 0.01) towards the IgG2a response in those injected with two doses of QIV compared with those injected with only one dose of the vaccine (Figure 7a).

Next, and considering the aforementioned changes in the IgG2a/IgG1 ratio following the second QIV injection, serum titres of IgG1 and IgG2a antibodies were determined against each of the four influenza virus strains from the vaccine in these mice. Following the second QIV injection, the supplementation with antioxidants/immunomodulators statistically significantly affected serum IgG1 titres specific to none of the influenza virus strains from QIV (Figure 7b). However, it increased the serum titres of IgG2a antibodies specific to all strains from QIV (*p* ≤ 0.05), but the increase in the serum titre of B/Yamagata-specific IgG2a antibodies did not reach statistical significance (Figure 7b). Accordingly, following the second QIV injection in mice supplemented with antioxidants/immunomodulators, irrespective of virus influenza strain specificity, the serum IgG2a/IgG1 response ratio was shifted (*p* ≤ 0.05) to the side of IgG2a response (Figure 7b).

**Figure 8:**
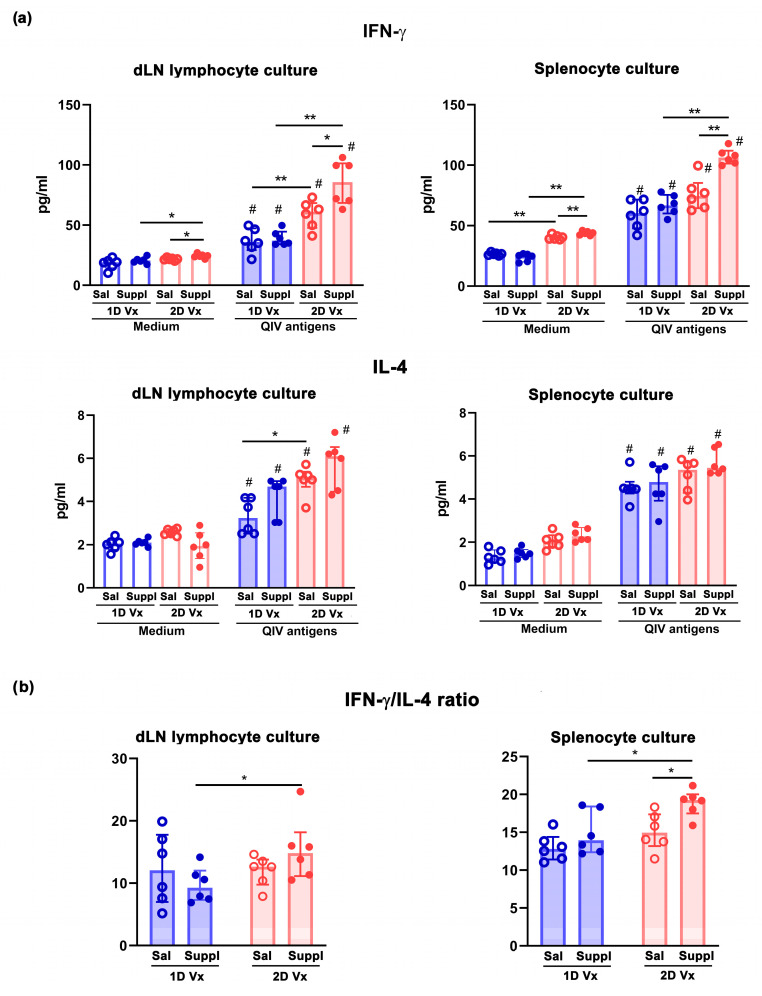
Supplementation with antioxidants/immunomodulators shifted IFN-γ/IL-4 secretion ratio towards IFN-γ in dLN and splenocyte cultures from mice injected with two doses of QIV. Bar graphs indicate (**a**) concentrations of IFN-γ and IL-4 and (**b**) IFN-γ/IL-4 ratio determined in cultures of drain lymph node (dLN) lymphocytes and splenocytes in the presence of QIV antigens (QIV antigens) and in medium alone (Medium). dLN lymphocytes and splenocytes were retrieved from mice administered with saline (Sal) or supplement (Suppl) 14 days after the first (1D) and the second (2D) dose of QIV (Vx). Data are presented as median and interquartile range (IQR) (individual data points are incorporated into bars). n = 6 mice/group. * *p* ≤ 0.05, ** *p* ≤ 0.01, # *p* ≤ 0.05 vs. Medium.

Given that Th immune response is crucial for shaping IgG subclass profile and thereby antibody functional capacity, where Th1 type cells elicit IgG2a antibody production and Th2 type cells induce IgG1 antibody secretion [26], in QIV-stimulated SLO lymphocyte cultures the production levels of IFN-γ and IL-4 (i.e., Th1 and Th2 cell signature cytokine, respectively) were measured [53]. Upon restimulation with QIV antigens, the levels of IFN-γ and IL-4 increased (*p* ≤ 0.01) in dLN cell cultures and splenocyte cultures over those in cultures without QIV antigens (negative controls) (Figure 8a). The level of IFN-γ did not statistically significantly differ between SLO cultures from mice injected with one dose of QIV and supplemented with antioxidant/immunomodulators, and cultures from mice injected with one dose of QIV and administered with saline (Figure 8a). On the other hand, in dLN and splenocyte cultures from mice injected with two doses of QIV, the level of IFN-γ was higher (*p* ≤ 0.05 and *p* ≤ 0.01 in dLN lymphocyte cultures and splenocyte cultures, respectively) in cultures from mice supplemented with antioxidants/immunomodulators than in those from mice administered with saline (Figure 8a). Additionally, it was also higher (*p* ≤ 0.01) in SLO cultures from mice injected with two doses of QIV and either supplemented with antioxidants/immunomodulators or administered with saline, compared with corresponding cultures from mice injected with one dose of the vaccine (Figure 8a). In SLO cultures from mice injected with either one dose of QIV or two doses of QIV, the level was comparable between cultures from mice supplemented with antioxidants/immunomodulators and cultures from mice administered with saline (Figure 8a).

Unlike IFN-γ level, the level of IL-4 influenced only immunization. It was higher in SLO cultures from mice injected with two doses of QIV compared with those from mice injected with one dose of the vaccine, but this increase reached statistical significance (*p* ≤ 0.01) only in dLN lymphocyte cultures (Figure 8a).

Additionally, considering that IFN-γ/IL-4 production level ratio, rather than absolute production level of any of two cytokines, may be considered as surrogate marker for IgG2a/IgG1 response ratio [53], their ratio was also examined. In cultures of cells from dLNs and spleens of mice injected with two doses of QIV, this ratio was shifted (*p* ≤ 0.05) towards IFN-γ (Figure 8b). In addition, it was shifted (*p* ≤ 0.01) towards IFN-γ more highly in SLO lymphocyte culture from supplemented mice injected with two doses of QIV compared with supplemented mice injected with one dose of the vaccine (Figure 8b).

**Figure 9:**
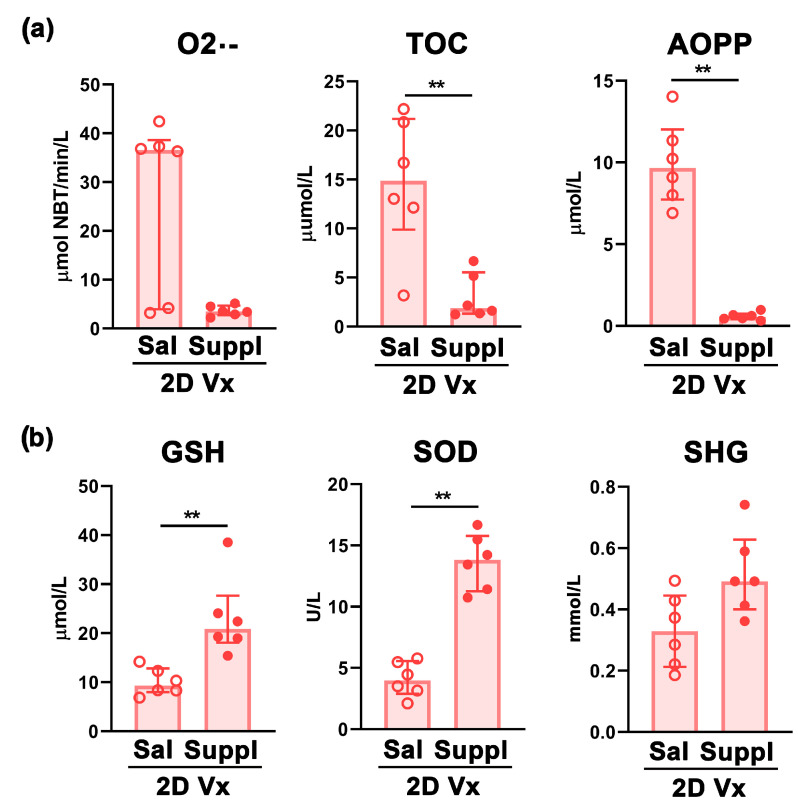
Supplementation with antioxidants/immunomodulators shifted the balance between prooxidant and antioxidant parameters towards the latter in spleens from mice injected with two doses of QIV. The bar graphs indicate the levels of: (**a**) pro-oxidant parameter superoxide anion radical (O2•−), total oxidant capacity (TOC), and parameter of tissue damage, i.e., advanced oxidation protein products (AOPP), and (**b**) antioxidant parameters, i.e., glutathione (GSH), Cu/Zn superoxide dismutase (SOD), and sulfhydryl groups (SHG) on the day 14 after the second QIV dose (2D Vx) in spleens of mice administered with saline (Sal) or supplement (Suppl). Data are presented as median and interquartile range (IQR) (individual data points are incorporated into bars). n = 6 mice/group. ** *p* ≤ 0.01.

Next, given that redox status is shown to influence Th cell polarization [54], to clarify the putative mechanism behind the shift in Th1/Th2 response in mice injected with two doses of QIV and supplemented with antioxidants/immunomodulators, the parameters of redox status in their spleens were investigated. Redox status was determined by measuring O2•−, AOPP, TOC, and several other antioxidant markers: SOD, SHG, and GSH, which are shown to have a central role in the maintenance of the thiol–disulfide redox state in mammalian cells [55]. Supplementation markedly diminished O2•− level in spleens from mice injected with doses of QIV, but this decrease did not reach statistical significance (Figure 9a). However, TOC was elevated (*p* ≤ 0.01) in spleens from mice supplemented with antioxidants/immunomodulators compared with mice administered with saline (Figure 9a). Consistently, AOPP level was markedly (*p* ≤ 0.01) lower in spleens from mice supplemented with antioxidants/immunomodulators compared with those administered with saline (Figure 9a). In accordance with these findings, compared with mice injected with two doses of QIV and administered with saline, in spleens from mice supplemented with antioxidants/immunomodulators and injected with two doses of QIV, the levels of all antioxidants were elevated, but only the changes in SOD and GSH reached statistical significance (*p* ≤ 0.01) (Figure 9b).

### 3.5. Antioxidant/Immunomodulatory Supplementation Increased Serum Neutralizing IgG Antibody Titre in Mice Injected with QIV

**Figure 10:**
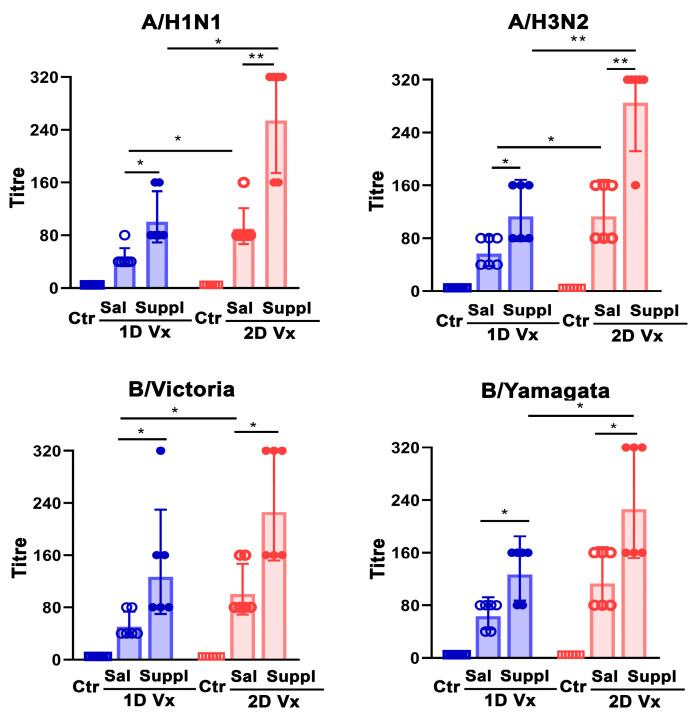
Supplementation with antioxidants/immunomodulators increased the titres of influenza virus-specific neutralizing antibodies. Graph bars indicate serum geometric mean titres of the neutralizing antibodies specific to influenza subtypes: A/H1N1, A/H3N2, B/Victoria lineage, and B/Yamagata lineage in mice injected with one dose (1D) and two doses (2D) of QIV (Vx), and treated with antioxidant/immunomodulatory supplement (Suppl) or saline (Sal), and in mice injected intramuscularly with Sal and treated per os with Suppl (control group, Ctr). Titres were determined 28 days after the first injection of QIV/Sal (1D) and 14 days after the second injection of QIV/Sal (2D). Error bars indicate the 95% confidence interval (CI). n = 6 mice/group. * *p* ≤ 0.05, ** *p* ≤ 0.01.

Given that it is widely accepted that the measure of the protection conferred by vaccination cannot solely rely on antibody magnitude, but also requires measurement of their functional properties [10], sera from QIV-immunized mice were also evaluated for the titres of neutralizing antibodies. The supplementation with antioxidants/immunomodulators increased the titres of neutralizing antibodies against all influenza virus strains incorporated in QIV in mice injected with one (*p* ≤ 0.05) and two (*p* ≤ 0.01 for A strains; *p* ≤ 0.05 for B strains) doses of the vaccine (Figure 10). Of note, in mice administered with saline, and following the second QIV injection, the titres of neutralizing antibodies against all influenza strains increased (*p* ≤ 0.05), but the increase in the titre of neutralizing antibodies against B (Yamagata) did not reach statistical significance (Figure 10). Accordingly, the titres of neutralizing antibodies against all influenza strains from QIV were greater in supplemented mice injected with two doses of QIV compared with their counterparts injected with only one dose of this vaccine, although the increase in titre of neutralizing antibodies B (Victoria) did not reach statistical significance (Figure 10).

## 4. Discussion

This study revealed that a supplement combining micronutrients with antioxidant/immunomodulatory properties (vitamins C and D, oligoelements zinc, manganese and selenium, and NAC), which are at high risk of deficit, influences humoral immune response to QIV. This supplement, administered to mice with one injection of QIV, increased (i) the serum titres of IgG and consistently the frequency of GC B cells in SLOs and, more important, (ii) the serum titres of neutralizing antibodies against each of four influenza virus strains from the vaccine. This could be at least partly associated with QIV-specific expansion of Tfh cells in their SLOs, a finding consistent with the increased frequency of IL-21+ T cells among SLO cells ex vivo and upon restimulation with QIV antigens in SLO cell cultures. On the other hand, the supplement in mice administered with two injections of QIV, increased the serum titres of neutralizing antibodies and IgG2a antibodies against all influenza strains in vaccine without significantly affecting the serum QIV-specific IgG titre (which was substantially increased 14 days following the second QIV injection in mice administered with saline). The latter could be ascribed to the shift in the Th1/Th2 signature cytokine-production level ratio towards Th1 cytokine (IFN-γ); the phenomenon reflecting at least partly the significant increase in antioxidant status in their SLOs.

To explain the reported increase in the serum influenza virus-specific IgG titres in response to the first QIV injection, an extensive survey of literature on the influence of each component of the supplement on antibody response to influenza vaccine/antiviral vaccines was carried out. The survey showed that, although all components of the supplement except for NAC may affect antibody synthesis and consequently serum IgG titres, data on significance of their supplementation/deficiency for humoral immune response to influenza vaccine (and thereby the vaccine efficacy/protective capacity), are inconclusive and inconsistent or lacking. Specifically, although zinc supplementation is shown to stimulate antibody synthesis [56,57], data from a rather limited number of studies investigating antibody responses to the influenza vaccine in relation to zinc status are controversial [58]. Namely, this is because they indicate that zinc supplementation may increase the humoral immune response to the influenza vaccine [59], but also that this supplementation is ineffective in this respect [60]. On the other hand, although it has been suggested that supplementation with vitamin D may diminish antibody response [61], there are data indicating this vitamin is ineffective in modulating antibody response, and that it may enhance antibody response in humans and mice [62,63,64], or at least this response to some influenza virus strains [65]. These discrepancies in findings obtained in studies on adjuvant capacities of zinc and vitamin D in influenza vaccination could be reconciled by differences in many factors that influence its outcome. Namely, participants in these studies differ in respect to many characteristics—to name a few: serum/plasma status of the supplement under investigation and, in studies involving humans, pre-vaccinated influenza virus-specific antibody levels [58,66]. Additionally, in vitamin D studies polymorphisms of gene for vitamin D receptors could also contribute to the outcome of the vaccination [58,66]. Moreover, supplement dose, its bioavailability, and regime of administration may also be contributing factors to the vaccination outcome [58,66]. Additionally, it has been shown that selenium may enhance antibody response to the influenza vaccine in mice and young adults by increasing glutathione peroxidase 4 level in Tfh cells, and thereby preventing their lipid peroxidation-induced ferroptosis [67]. The stimulatory effect of selenium on antibody response to some other vaccines has also been shown [68,69]. Furthermore, there are data on the stimulatory influence of vitamin C [70,71,72,73] and manganese [74,75] on antibody synthesis in response to immunization with some other T-dependent antigens, including vaccinal antigens, in humans and/or experimental animals.

The increase in the serum QIV-specific IgG and neutralizing antibody titres 28 days after the first immunization in mice supplemented with antioxidants/immunomodulators, compared with their counterparts administered with saline, was in accordance with the higher frequency of GC B cells in their SLOs. This finding could be ascribed to their greater proliferative response, as it was shown in QIV-restimulated SLO cell cultures. The greater proliferative response of GC B cells from supplemented mice compared with their saline-administered counterparts was consistent with the higher frequency of Tfh cells, which provide CD40L and IL-21 signals required for proliferation and differentiation of B cells [76], reflecting partly lower Tfr/Tfh cell ratio in their SLOs [77]. Additionally, it was also in accordance with lower Tfr/GC B cell ratio in their SLOs in mice administered with antioxidants/immunomodulators compared with their saline- administered counterparts [77]. It is noteworthy that the supplementation did not significantly affect the frequency of Tfr cells on the 14th day after immunization. Data on the kinetics of changes in Tfr cell number following influenza virus infection corroborate this finding [78]. Namely, they indicate that the rise in Tfr cell number requires time peaking at 30 days following the infection [78]. More important, it has been suggested that Tfr cells control different aspects of humoral immune response to foreign antigens at specific stages of the GC reaction, viz., they have a major influence on the magnitude of antibody response to foreign antigens at early stages of the immune response, whereas at later stages (e.g., 36 days after influenza infection and beyond) they control the specificity of the antibody response [77,78,79,80]. The higher frequency of Tfh cells in SLOs from QIV-immunized mice supplemented with antioxidants/immunomodulators is consistent with the greater proliferative response of their GC CD4+CXCR5+ cells upon stimulation with QIV antigens in cultures compared with those cells from their counterparts administered with saline. In favor of these findings are data indicating that vitamin C [81,82,83], zinc [56,57,84], and selenium [69,85] stimulate GC T-cell and B-cell differentiation and proliferation, leading to enhanced antibody generation. The promotion of T cell expansion and survival by IL-21 is shown to set the GC response magnitude, and variation in either its production or sensitivity to its action may alter the outcomes of humoral immune response [25]. This is reported to increase the frequency of IL-21-producing T-cells (the major source of IL-21 in SLOs) among SLO cells from mice supplemented with antioxidants/immunomodulators and injected with one dose of QIV ex vivo and in vitro upon restimulation with QIV, compared with their saline-administered counterparts, and may point to a putative mechanism of stimulatory action of administered supplements on T-cell proliferation and survival. To the best of our knowledge there are no data to directly corroborate the influence of antioxidants/immunomodulators used for supplementation on IL-21+ T cell differentiation, and consequently antibody response. However, supplementary vitamin C has been identified as a potent enhancer for antibody response by directly facilitating action on the IL-21/STAT3-dependent plasma cell differentiation in mice and humans [86,87].

To explain at least partly the lack of stimulatory effects of antioxidant/immunomodulatory supplementation on GC T cell and GC B cell proliferation, and consequently the serum QIV-specific IgG titres in mice administered with two injections of QIV, are data indicating that the proliferative response of T cells to supplementation with antioxidants/immunomodulators, such as zinc, depends on the starting activation status of the lymphocytes. So, in low responders this was enhanced, whereas, in high responders it tended to be reduced [57], most likely implying a differential response of QIV-specific naïve and memory cells, i.e., cells predominantly responding upon primary and secondary QIV immunization, respectively.

Although in mice injected with two doses of QIV the serum IgG titres were comparable between animals supplemented with antioxidants/immunomodulators and those administered with saline, in supplemented animals, as in their counterparts injected with one dose of QIV, the serum titre of neutralizing antibody was greater than in mice administered with saline. Given that antibodies must be specific for the variant virus, as the neutralizing epitopes differ between strains [88], it should be emphasized that titres of neutralizing antibodies specific to each of the four influenza strains from QIV were greater in mice supplemented with antioxidants/immunomodulators, compared with saline-administered ones. To corroborate the lack of changes in the serum IgG titres, the Tfr/Tfh and Tfr/GC B ratios were comparable between animals supplemented with antioxidants/immunomodulators and their counterparts administered with saline [77]. Considering the supplement-induced rise in the serum titres of neutralizing antibodies, it is noteworthy that, unlike the initial belief that Tfr cells represent the regulatory counterpart for Tfh cells in the GC reaction, an accumulating body of evidence indicates that Tfr cells are predominantly involved in the fine tuning of antibody response to foreign antigens mirrored in the promotion of antigen-specific GC B cell responses, i.e., in focusing the antibody specificity towards the immunizing antigen while avoiding the emergence of self-reactive antibodies [77]. More relevantly, it was discovered that, following influenza virus challenge, Tfr cells are necessary for the robust generation of virus-specific, long-lived plasma cells and antibody production against both HA and neuraminidase, the two major influenza virus glycoproteins, and for the appropriate regulation of the B-cell receptor repertoire and the development of long-term humoral memory [78]. Indeed, in mice administered with saline, following the second injection of QIV, the relatively greater rise in the number of Tfr cells compared with the rise in the number of Tfh and GC B cells was found, and consequently the Tfr/Tfh and Tfr/GC B cell ratios were shifted towards Tfr cells. Consistently, in these mice the serum titres of neutralizing antibodies were greater in those who received two injections of QIV. However, although the serum titres of neutralizing antibodies were greater in supplemented mice administered with two injections of QIV than in their counterparts receiving saline, Tfr/Tfh and Tfr/GC B cell ratios in their SLOs were comparable. Considering that the net effect of Tfr cell regulatory (“helper”) action depends not only on their number but also on the efficacy of their action, it may be speculated that supplementation affects the Tfr effector mechanism (CTLA-4 and, more recently, neuritin and fibrinogen-like protein-2) or the target cell sensitivity to their action [77].

It is also of note that, although IgG2a and IgG1 antibodies are endowed with neutralization properties, IgG2a antibodies are of special value in the protection against infection due to their potent Fc biologic effector functions, such as antibody-dependent cellular cytotoxicity and antibody-dependent phagocytosis [89,90,91,92]. In this context it should be emphasized that, in mice supplemented with antioxidants/immunomodulators and administered with two injections of QIV, the ratio of titres of IgG2a/IgG1 specific to antigens of each influenza virus strain from QIV were shifted towards IgG2a when compared with their counterparts administered with saline. Given that the ratio in the production levels of IFN-γ (Th1 signature cytokine) and IL-4 (Th2 signature cytokine) are critical for shaping the IgG2a/IgG1 ratio [53,93], the reported changes in their production level ratio upon restimulation with QIV in SLO cell cultures from supplemented mice who received two doses of QIV compared with cultures from their counterparts administered with saline corroborate the shift in the serum QIV-specific IgG2a/IgG1 ratio. Considering that oxidative stress promotes the shift in Th1/Th2 cell ratio towards the latter [54], the shift in pro-oxidant/antioxidant status towards the latter, leading to a diminished generation of AOPP in spleens from mice injected with two doses of QIV and supplemented with antioxidants/immunomodulators compared with their counterparts administered with saline, additionally corroborates the previous findings. In the same line are data indicating that vitamin C [87,94], zinc [95,96,97], and selenium [85,98] are shown to promote isotype switching to IgG2a in mice and/or humans in a redox status independent manner. Additionally, vitamin D, which is also well-known for its antioxidant properties [99,100,101], is acknowledged to shift the Th response profile from a Th1 to a Th2-mediated response through an oxidative stress-independent action [99,100]. Thus, it may be assumed that this study’s reported supplementation-induced changes in Th1/Th2-signature-cytokine-production-level ratio represent the net effect of the antioxidant action of antioxidants used for the supplementation, and their direct antioxidant-independent action on Th cell differentiation. Thus, the analysis of IgG subclass profile in conjunction with the analysis of serum-neutralizing antibody titre suggests that that continuous supplementation with antioxidants/immunomodulators following primary vaccination leads to a more effective protective immune response to subsequent challenges by antigens from the influenza vaccine.

## 5. Conclusions

In conclusion, briefly, the supplementation with vitamins D and C, oligoelements zinc, selenium and manganese, and modified amino acid cysteine NAC (i) augmented the magnitude of the primary serum IgG and, particularly importantly, neutralizing antibody titres by stimulating GC reaction, and (ii) optimized the secondary IgG antibody response by stimulating more favorable (viz., more effective in the context of virus infection clearance) IgG2a response. The latter could be ascribed to its direct and indirect stimulatory action (through modulation of the provident/antioxidant balance) on Th1 cell differentiation and, consequently, Th1/Th2 cell balance in SLOs. Thus, the study provided a solid base for further studies aimed at repurposing the use of this supplement combination to be used not only to prevent virus infection and stimulate recovery, but also to improve quality of immune responses to an inactivated influenza virus vaccine and possibly some other virus vaccines. To add additional weight to this notion are data indicating that nutritional gaps in several micronutrients reported to support immune function, including vitamins A, D, and E, as well as iron, zinc, and selenium are, relatively, more highly prevalent [22]. Thus, supplementation combining the micronutrients at highest risk of deficit should be considered a safe and effective way to prevent or correct inadequacies, and to reduce the incidence and severity of infective diseases.

## Data Availability

Data is contained within the article.
